# *Pantoea alhagi*, a novel endophytic bacterium with ability to improve growth and drought tolerance in wheat

**DOI:** 10.1038/srep41564

**Published:** 2017-01-27

**Authors:** Chaoqiong Chen, Kaiyun Xin, Hao Liu, Juanli Cheng, Xihui Shen, Yao Wang, Lei Zhang

**Affiliations:** 1 State Key Laboratory of Crop Stress Biology for Arid Areas and College of Life Sciences, Northwest A&F University, Yangling, Shaanxi 712100, PR China; 2 Life Sciences Department, Yuncheng University, Yuncheng 044000, PR China

## Abstract

A novel strain LTYR-11Z^T^ that exhibited multiple plant growth promoting (PGP) traits was isolated from the surface-sterilized leaves of *Alhagi sparsifolia* Shap. (*Leguminosae*), which reprsents one of the top drought tolerant plants in north-west China. Phylogenetic analysis of 16S rRNA gene sequences and multilocus sequence analysis based on partial sequences of *atpD, gyrB, infB* and *rpoB* genes revealed that strain LTYR-11Z^T^ was a member of the genus *Pantoea*, with *Pantoea theicola* NBRC 110557^T^ and *Pantoea intestinalis* DSM 28113^T^ as the closest phylogenetic relatives. The results of DNA–DNA hybridization, phenotypic tests and fatty acid analysis confirmed that strain LTYR-11Z^T^ represents a novel species of the genus *Pantoea*, for which we propose the name *Pantoea alhagi* sp. nov. Confocal microscopy observation revealed that strain LTYR-11Z^T^ effectively colonizes the rhizoplane of both *Arabidopsis* and wheat. Strain LTYR-11Z^T^ was able to promote the growth of wheat enhancing its resistance to drought stress. Strain LTYR-11Z^T^ led to increased accumulation of soluble sugars, decreased accumulation of proline and malondialdehyde (MDA), and decreased degradation of chlorophyll in leaves of drought-stressed wheat. Our findings will contribute to the development of a novel biotechnological agent to improve the adaptation of crop plants to drought in arid ecosystems.

Plant growth promoting (PGP) bacteria are found to inhabit plant surfaces and different plant tissues and organs, including the rhizosphere, phyllosphere, roots, stems, leaves, flowers, seeds and fruits^1^,^2^. Endophytes may be of particular interest as they can colonize internal tissues and are thus relatively protected from the competitive and high-stress soil environment^1^. It is now well recognized that each individual plant, and nearly all tissues within the plant, is host to one or more endophytes^3^. In recent years, bacterial endophytes have received considerable attention for their ability to improve plant tolerance against abiotic stresses such as salinity, drought, cold and heavy metal toxicity^4^,^5^,^6^. It has been reported that PGP bacteria can contribute to alleviate abiotic stresses of host plants via a variety of mechanisms^3^,^4^. Lowering of ethylene level by 1-aminocyclopropane-1-carboxylate (ACC) deaminase is considered to be one of the major mechanisms employed by PGP bacteria to favor plant growth under stress conditions^5^,^7^. The production of exopolysaccharides (EPS) can also play an important role in supporting plant growth under water deficit and salt stress conditions^8^,^9^. Other metabolites produced by PGP bacteria and potentially involved in supporting plant growth under stress conditions include siderophores, phytohormones, organic acids and volatile compounds^8^,^10^. In addition, PGP bacteria may enhance plant stress tolerance by inducing accumulation of osmolytes, antioxidants, regulation of stress responsive genes, increase in photosynthesis and alteration in root morphology^10^,^11^.

Drought is one of the major environmental stresses that limit crop growth and productivity worldwide, while global warming and water scarcity will further make the situation worse^10^. Thus, it is urgent to develop crop plants with improved drought tolerance^3^,^12^. Several recent ecological studies have found that microbial symbionts can confer habitat-specific stress tolerance to host plants, suggesting that the basis for the stress tolerance-enhancing effects of microbial symbionts is based on the co-evolution of plant and microbes under harsh environmental conditions^13^,^14^,^15^. Thus, it is a good strategy to look for plant-beneficial microorganisms that confer resistance to a specific environmental stress from the soil environments where that stress is a regular phenomenon^3^. *Alhagi sparsifolia* Shap. (*Leguminosae*), a typical desert plant species, is widely distributed in the arid and salinized regions of north-west China. This perennial plant has highly developed deep roots and possesses a great capacity to withstand poor soil and severe drought conditions, thereby playing a fundamental role in maintaining the desert ecosystem^16^. Therefore, *A. sparsifolia* may represent an ideal species for discovering novel biotechnological agents for use in arid land agriculture. On this background, *A. sparsifolia* was collected from the Taklamakan Desert, Xinjiang Uyghur Autonomous Region, north-west China, and endophytic bacterial communities from its leaves, stems and roots were isolated and subjected to *in vitro* tests for PGP traits and abiotic stress tolerance. A novel bacterial strain, designated LTYR-11Z^T^, which was isolated from the surface-sterilized leaves of *A. sparsifolia*, exhibited multiple PGP traits and stress resistance capabilities, including mineral phosphate solubilization, production of indole-3-acetic acid (IAA), siderophores, EPS and protease, and tolerance to osmotic stress, salt and high temperature. Phylogenetic analysis of nearly full-length 16S rRNA gene sequences and a concatenate of partial *gyrB, rpoB, infB* and *atpD* gene sequences indicated that strain LTYR-11Z^T^ should be assigned to the genus *Pantoea*.

The genus *Pantoea* was first described by Gavini *et al*.^17^, with *Pantoea agglomerans* as the type species. Currently, this genus comprises 22 species with validly published names (http://www.bacterio.net/pantoea.html). Species of the genus *Pantoea* are Gram-negative, facultatively anaerobic, non-spore-forming rods and commonly motile by means of peritrichous flagella^18^. Members of the genus *Pantoea* have been isolated from various environmental habitats such as soils, water, foods, plants, humans and other animals^19^,^20^,^21^. Some *Pantoea* species are known to interact with plants and may confer beneficial or deleterious effects to their hosts^22^,^23^. For example, *P. agglomerans* was initially identified as a cause of diseases in a wide range of hosts, such as onion, gypsophila, beet and wisteria, beach pea, cotton, rice, maize^17^,^19^,^23^, but many strains belonging to this species also occur as plant epiphytes or endophytes and exhibit PGP or biocontrol effects^24^,^25^.

Here we report on the taxonomic characterization of this newly isolated *Pantoea* sp. LTYR-11Z^T^ by using a polyphasic approach. Phenotypic, genotypic and phylogenetic data revealed that strain LTYR-11Z^T^ represents a novel species of the genus *Pantoea*, for which the name *Pantoea alhagi* sp. nov. is proposed. The role of this novel species in growth promotion as well as drought stress alleviation in wheat plants was also assessed by *in vivo* studies. Our results showed that strain LTYR-11Z^T^ successfully colonized the root system of wheat plants, enhanced plant growth and improved plant response to drought stress. The discovery of the drought resistance-promoting bacterium *Pantoea alhagi* sp. nov. LTYR-11Z^T^ will be helpful for the further investigations into the mechanisms behind bacteria-mediated drought tolerance in plants.

## Results

### Phylogenetic analysis based on 16S rRNA, *atpD, gyrB, infB* and *rpoB* gene sequences

**Figure 1:**
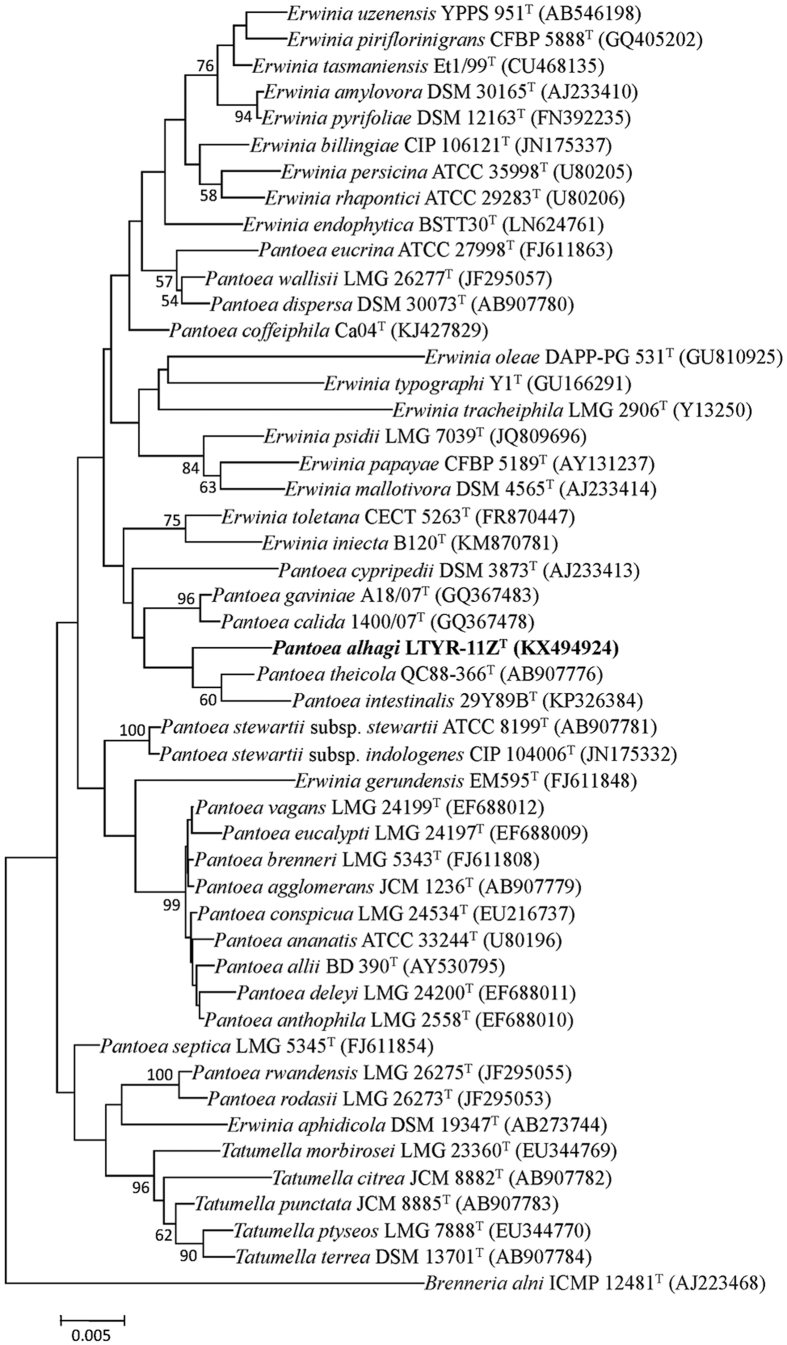
Neighbour-joining phylogenetic tree based on 16S rRNA gene sequences showing the relationships between strain LTYR-11Z^T^ and related type strains of the genus *Pantoea, Erwinia* and *Tatumella*. Numbers at nodes indicate bootstrap percentages (based on 1000 resampled datasets), and only values above 50% are shown. *Brenneria alni* ICMP 12481^T^ was used as an outgroup. Bar, 0.005 substitutions per nucleotide position.

The nearly complete 16S rRNA gene sequence of strain LTYR-11Z^T^ (1464 bp; GenBank/EMBL/DDBJ accession number KX494924) was obtained and subjected to comparative analysis. On the basis of 16S rRNA gene sequence similarities, the closest related species of strain LTYR-11Z^T^ were *Pantoea theicola* NBRC 110557^T^ (98.7%), *Pantoea intestinalis* DSM 28113^T^ (97.7%), *Erwinia gerundensis* EM595^T^ (97.6%), *Erwinia uzenensis* YPPS 951^T^ (97.5%), *Erwinia piriflorinigrans* CFBP 5888^T^ (97.4%), *Erwinia pyrifoliae* DSM 12163^T^ (97.4%) and *Pantoea gaviniae* LMG 25382^T^ (97.4%). Sequences similarities of less than 97.3% were found with the type strains of other species of the genera *Pantoea* and *Erwinia*. In both neighbour-joining (NJ) and maximum-likelihood (ML) phylogenetic trees based on 16S rRNA gene sequences (Fig. 1, Fig. S1), *Pantoea* and *Erwinia* species were shown to be polyphyletic, which is in agreement with previous reports^20^,^21^. Nevertheless, strain LTYR-11Z^T^ was found to form a coherent cluster with *P. theicola* NBRC 110557^T^ and *P. intestinalis* DSM 28113^T^, suggesting that strain LTYR-11Z^T^ belongs to the genus *Pantoea*.

**Figure 2:**
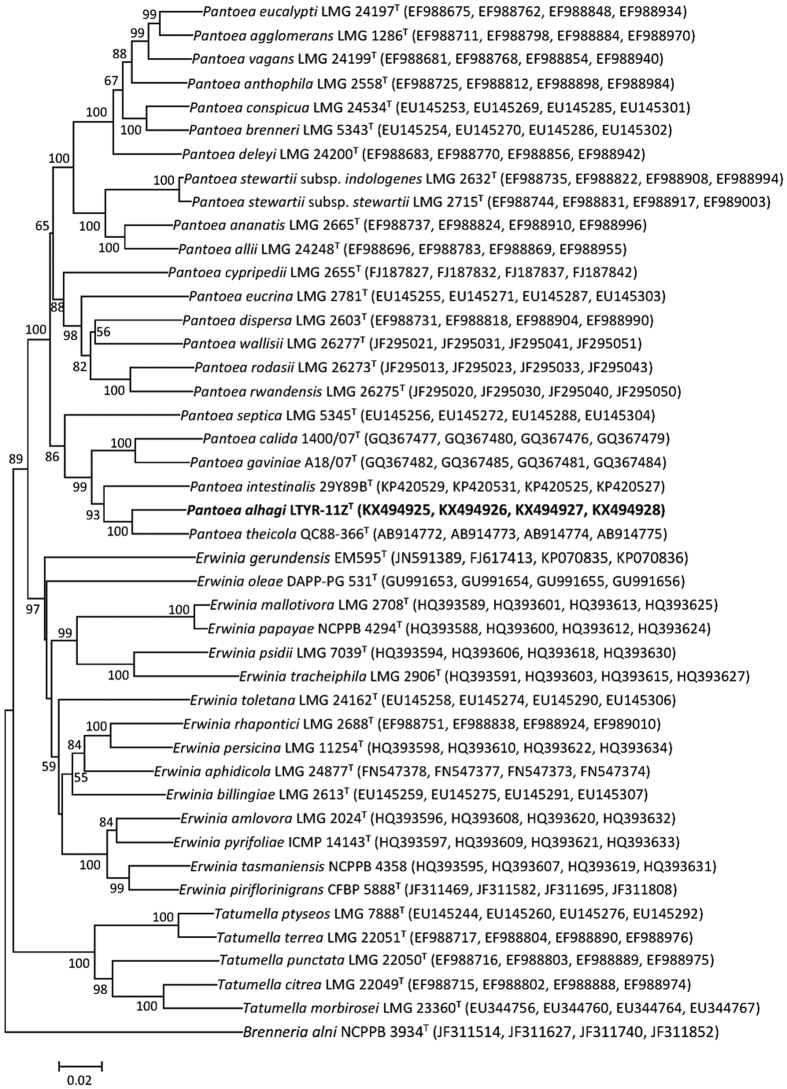
Neighbour-joining phylogenetic tree based on concatenated partial sequences of *atpD, gyrB, infB* and *rpoB* gene sequences showing the relationships between strain LTYR-11Z^T^ and related species of the genus *Pantoea, Erwinia* and *Tatumella*. Numbers at nodes indicate bootstrap percentages (based on 1000 resampled datasets), and only values above 50% are shown. *Brenneria alni* NCPPB 3934^T^ was used as an outgroup. Bar, 0.02 substitutions per nucleotide position.

Multilocus sequence analysis (MLSA) of concatenated partial *atpD, gyrB, infB* and *rpoB* gene sequences has been proved to be a useful tool for differentiating the phylogenetically related genera *Erwinia, Pantoea* and *Tatumella* from each other^19^,^20^. To define the taxonomic position of strain LTYR-11Z^T^, the partial *atpD, gyrB, infB* and *rpoB* gene sequences of strain LTYR-11Z^T^ (850, 923, 1082 and 648 bp, respectively; GenBank/EMBL/DDBJ accession numbers KX494925-KX494928) were determined and the NJ and ML trees based on concatenation of these four gene sequences were constructed (Fig. 2, Fig. S2). In both trees, the genera *Pantoea* and *Erwinia* were shown to be monophyletic, while strain LTYR-11Z^T^ fell within the clade comprising *Pantoea* species, forming a tight phyletic group with *P. theicola* NBRC 110557^T^ and *P. intestinalis* DSM 28113^T^ with high bootstrap values (>92%). Thus, MLSA analysis of concatenated partial *atpD, gyrB, infB* and *rpoB* gene sequences combining 16S rRNA gene sequence analysis revealed that strain LTYR-11Z^T^ is a member of the genus *Pantoea* and its closest phylogenetic relatives are *P. theicola* NBRC 110557^T^ and *P. intestinalis* DSM 28113^T^. Strain LTYR-11Z^T^ exhibited MLSA sequence similarity of 94.8 and 91.9% to *P. theicola* NBRC 110557^T^ and *P. intestinalis* DSM 28113^T^, respectively, and of less than 91.4% to the type strains of other species of the genus *Pantoea*. These values were lower than 96.4%, the recommonded threshold of MLSA sequence similarity for division of *Pantoea* species^20^, suggesting that strain LTYR-11Z^T^ represents a novel species of the genus *Pantoea*.

### Genomic DNA G+C content and DNA–DNA hybridization

The DNA G+C content of strain LTYR-11Z^T^ was 53.4 mol%, which is within the range reported for species of the genus *Pantoea* (52.7–60.6 mol%)^18^, but much lower than those of its closest phylogenetic relatives *P. theicola* NBRC 110557^T^ and *P. intestinalis* DSM 28113^T^ (57.2 and 59.1mol%, respectively)^20^,^26^. Levels of DNA–DNA relatedness between strain LTYR-11Z^T^ and *P. theicola* NBRC 110557^T^ and *P. intestinalis* DSM 28113^T^ were 44.2 ± 6.6% and 32.1 ± 4.7%, respectively, clearly lower than the threshold value of 70% recommended to be used as a standard for species delineation^27^.

### Phenotypic characteristics of *Pantoea alhagi* LTYR-11Z^T^

Cells of strain LTYR-11Z^T^ were Gram-negative, facultatively anaerobic, motile short rods, with 1.3–2.1 μm in length and 0.7–0.9 μm in width (Fig. S3). Strain LTYR-11Z^T^ grew well on trypticase soy agar (TSA), LB agar, nutrient agar (NA) and R2A agar, and moderately on MacConkey agar (all from Difco). Growth was found to occurr at 7–48 °C (optimum, 37 °C), at pH 5.0–9.0 (optimum, pH 7.0) and in the presence of 0–9% (w/v) NaCl (optimum, 0–2%). Strain LTYR-11Z^T^ was sensitive to chloramphenicol, kanamycin, neomycin sulfate, streptomycin, tetracycline and vancomycin (50 μg ml^−l^), but was resistant to ampicillin (100 μg ml^−l^). Strain LTYR-11Z^T^ was positive for hydrolysis of aesculin, carboxymethyl cellulose, casein and gelatin, but negative for hydrolysis of chitin, soluble starch and L-tyrosine. Strain LTYR-11Z^T^ could be differentiated from the most closely related type strains *P. theicola* NBRC 110557^T^ and *P. intestinalis* DSM 28113^T^ by a range of phenotypic properties (Table 1). In contrast to its phylogenetically closest neighbour *P. theicola* NBRC 110557^T^, strain LTYR-11Z^T^ was positive for ampicillin resistance (50 and 100 μg ml^−l^), hydrolysis of casein and gelatin, growth at 45 °C and in the presene of 9% NaCl, indole production, Voges–Proskauer reaction (acetoin production), assimilation of malic acid, and acid production from D-sorbitol, sucrose and D-lactose, and was negative for *α*-glucosidase and *N*-acetyl-*β*-glucosaminidase activities, and acid production from salicin and D-fucose.

### Fatty acid analysis

The major cellular fatty acids in strain LTYR-11Z^T^ were identified as C_16:0_ (25.3%), summed feature 3 (comprising C_16:1_*ω*7*c* and/or C_16:1_*ω*6*c*; 21.8%), C_17:0_ cyclo (11.9%), summed feature 2 (comprising any combination of C_12:0_ aldehyde, an unknown fatty acid of equivalent chain length 10.928, iso-C_16:1_ I and C_14:0_ 3-OH; 11.0%), summed feature 8 (comprising C_18:1_*ω*7*c* and/or C_18:1_*ω*6*c*; 9.5%), C_12:0_ (8.8%) and C_14:0_ (6.3%). The fatty acid profile of LTYR-11Z^T^ was essentially similar to those of closely related phylogenetic neighbours *P. theicola* NBRC 110557^T^ and *P. intestinalis* DSM 28113^T^, which also contained C_16:0_ (32.5–34.9%), summed feature 3 (8.6–15.4%), C_17:0_ cyclo (10.8–15.2%), summed feature 8 (13.1–14.3%), summed feature 2 (9.0–12.2%), C_14:0_ (6.5–8.4%) and C_12:0_ (4.2–5.9%) as their major fatty acids (Table 2). Nevertheless, clear differences in the fatty acid profiles of strain LTYR-11Z^T^ and the two reference strains could be observed. The presence of iso-C_17:0_ 3-OH, the absence of iso-C_19:0_ together with significant differences in the proportions of C_16:0_, summed feature 3 and summed feature 8 distinguished strain LTYR-11Z^T^ clearly from its closest phylogenetic relative *P. theicola* NBRC 110557^T^ (Table 2).

### *In vitro* PGP activities and abiotic stress tolerance

Strain LTYR-11Z^T^ was able to produce IAA (17.73 ± 2.86 μg ml^−1^), siderophores, EPS, protease and ammonia. Strain LTYR-11Z^T^ was found to possess mineral phosphate solubilization activity by the appearance of phosphate-solubilizing halo on NBRIP plates. After 4 days of incubation, the concentration of soluble P in the NBRIP supernatant reached 1525.2 ± 37.2 mg L^−1^. In addition, strain LTYR-11Z^T^ was able to grow at 7 °C and 48 °C, in tryptic soy broth (TSB) supplemented with 20% polyethylene glycol (PEG) (molecular weight, 6000) and tolerated up to 9% NaCl. However, the ACC deaminase activity was not detected in strain LTYR-11Z^T^.

### Root colonization

**Figure 3:**
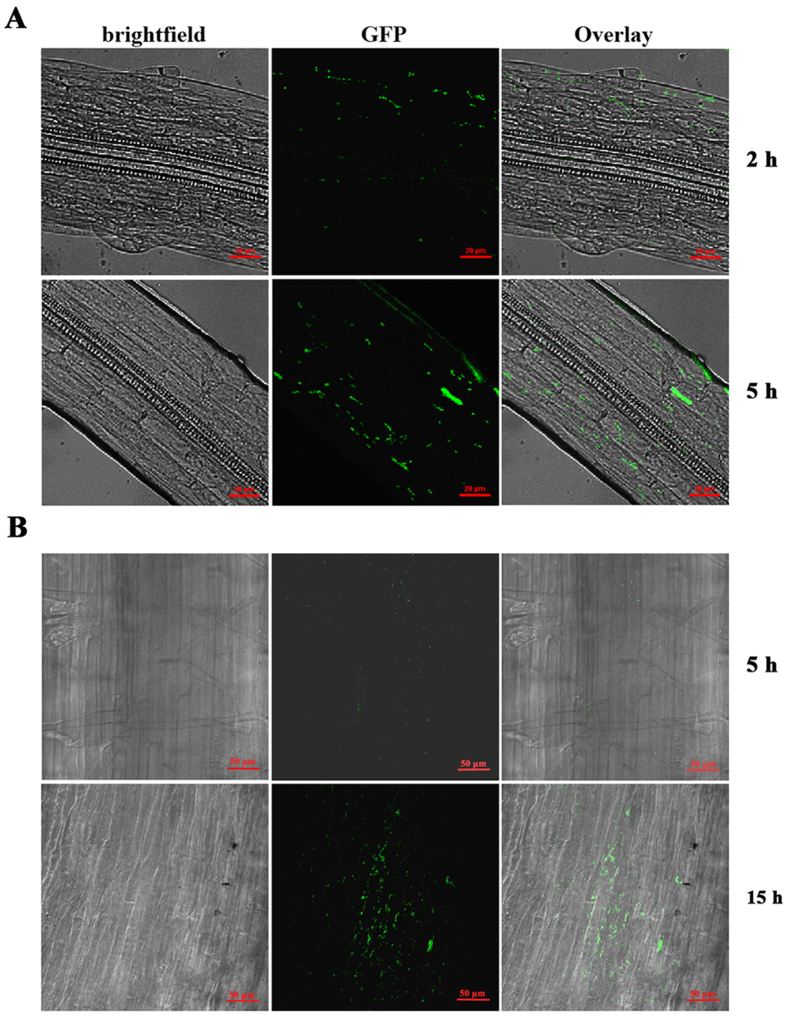
Confocal Laser Scanning micrographs of *Arabidospis thaliana* and wheat roots colonized by the GFP-labelled strain LTYR-11Z^T^. (**A**) Colonization of *Arabidospis thaliana* rhizoplane after 2 h and 5 h of exposure to the GFP-labelled strain LTYR−11Z^T^. (**B**) Colonization of wheat rhizoplane after 5 h and 15 h of exposure to the GFP-labelled strain LTYR-11Z^T^. The scale bars correspond to 20 μm in (**A**) and 50 μm in (**B**).

To assess the ability of strain LTYR-11Z^T^ to adhere and colonize the rhizoplane, an adhesion assay was performed on *Arabidopsis thaliana* and wheat roots by using the GFP-labelled bacterial cells. After 2 h of incubation in the bacterial suspension of GFP-labelled LTYR-11Z^T^, confocal microscopy observation revealed the presence of a small number of GFP-labeled cells adhering to the *Arabidopsis* root surface (Fig. 3A). After a longer incubation of 5 h, the *Arabidopsis* rhizoplane was massively colonized by the GFP-tagged bacterial cells, which were largely aggregated in the longitudinal sections of the roots (Fig. 3A). Likewise, root colonization by the GFP-labelled LTYR-11Z^T^ was also observed on wheat roots. After 5 h of exposure to the GFP-labelled bacterial suspension, wheat root surface was colonized with a few scattered GFP-expressing cells (Fig. 3B). Nevertheless, fifteen hours after incubation, GFP-labelled LTYR-11Z^T^ were found to colonize the wheat rhizoplane at high population levels (Fig. 3B). These results confirmed the ability of strain LTYR-11Z^T^ to efficiently colonize the roots of both *Arabidopsis* and wheat.

### Strain LTYR-11Z^T^ improves plant growth and drought tolerance

**Figure 4:**
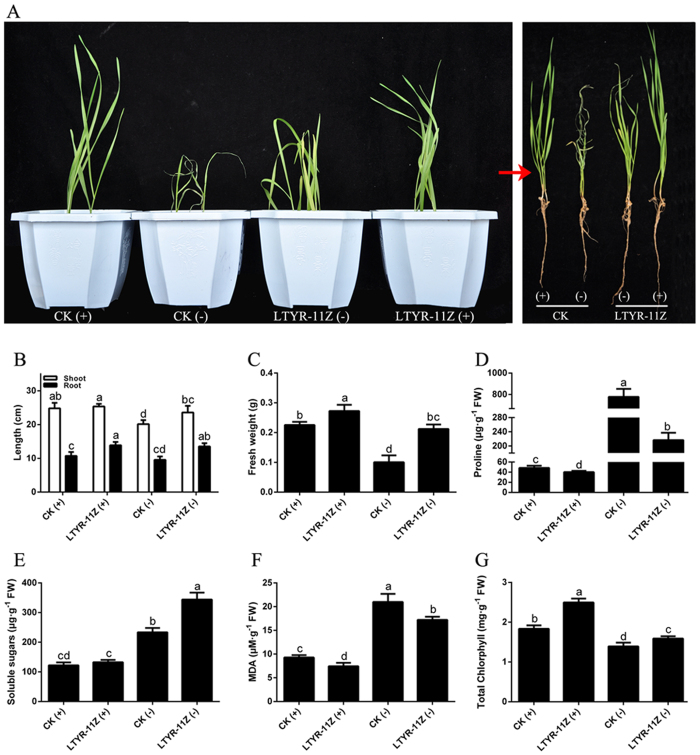
Strain LTYR-11Z^T^ promoted wheat growth and improved wheat resistance to drought stress. CK, non-inoculated control. ‘(+)’, irrigated at the water-holding capacity of the soil throughout the experiment; ‘−’, grown without water for 7 days and rewatered for 1 days. (**A**) Representative images of plants inoculated with strain LTYR-11Z^T^ compared to non-inoculated plants under well-irrigated and water stress conditions. (**B**) Shoot and root length. (**C**) Plant fresh weight. (**D**–**G**) Respectively represent the contents of free proline, soluble sugars, MDA and total chloropyhll in the wheat leaves. The data are means of three independent experiments. Error bars indicate standard deviation based on 3 replicates; every replicate contains 9 individual plants per treatment. Statistical significance was determined by one-way analysis of variance (ANOVA) followed by Brown-Forsythe test; different lower case letters indicate significant differences (P < 0.05) for each parameter.

On the basis of the multiple PGP traits, stress resistance capabilities and efficient root colonization ability of strain LTYR-11Z^T^, we further studied the effects of strain LTYR-11Z^T^ on the growth and drought resistance of wheat plants using pot experiments (Fig. 4). In the absence of stress, there were no obvious differences in the development and health of non-inoculated control and LTYR-11Z^T^-treated plants (Fig. 4A). However, increased root length (29.3%) and fresh weight (20.8%) were observed in plants inoculated with LTYR-11Z^T^ as compared to the non-inoculated control (Fig. 4B,C). After seven days of water deprivation, non-inoculated control plants became severely wilted and desiccated, whereas inoculated plants were not severely affected, with only slight curling of the leaf tips (Fig. 4A). Plants inoculated with LTYR-11Z^T^ showed 17.1%, 41.8% and 112% increase in shoot length, root length and total plant fresh weight, respectively, as compared to the non-inoculated stressed control (Fig. 4B,C). These results indicate that inoculation with LTYR-11Z^T^ has a positive impact on plant growth in the presence and absence of water stress, suggesting this novel isolate plays a dual role in growth enhancement and water stress tolerance.

In order to evaluate how strain LTYR-11Z^T^ promoted plant growth and alleviated water stress, physiological parameters including proline, soluble sugars, malondialdehyde (MDA) and chlorophyll contents were measured. Under non-stressed conditions, plants inoculated with LTYR-11Z^T^ accumulated lower levels of proline and MDA, equivalent levels of soluble sugars, but higher levels of chlorophyll in the leaves compared to the non-inoculated control plants (Fig. 4D–G). After exposure to water stress, the contents of proline, soluble sugars and MDA were significantly increased, while the chlorophyll contents were drastically decreased (Fig. 4D–G). However, inoculation with LTYR-11Z^T^ have positive or negative effects on these physiological parameters in drought-stressed plants. There was an increase of 47.5% and 14.4% respectively in the contents of soluble sugars and chlorophyll in drought-stressed plants inoculated with LTYR-11Z^T^ as compared to the non-inoculated stressed control (Fig. 4E,G). In contrast, the concentrations of proline and MDA in the inoculated plants showed 72.1% and 15.9% decrease, respectively, compared to the non-inoculated stressed control plants (Fig. 4D,F).

Re-isolation experiments from the surface-sterilized roots of the non-stressed wheat plants inoculated with LTYR-11Z^T^ showed a density of 2.5 × 10^6^ ± 8.4 × 10^5^ CFU g^−1^ fresh weight (FW), confirming the endophytic colonization of wheat roots by strain LTYR-11Z^T^. Interestingly, the re-isolation counts in the inoculated wheat plants exposed to water stress increased to 3.9 × 10^7^ ± 1.2 × 10^7^ CFU g^−1^ FW, suggesting that strain LTYR-11Z^T^ exhibits a stronger colonization activity upon water deficit stress.

Taken together, these data demonstrate that strain LTYR-11Z^T^ can efficiently colonize the root system of wheat seedlings, promote plant growth and improve plant tolerance against water stress.

## Discussion

Since drought has emerged as one of the most common environmental abiotic stresses that reduce crop production and productivity globally, creation of drought-tolerant cultivars by means of conventional breeding techniques and genetic engineering is widely used to mitigate the negative effects of drought stress on crop growth and yields^28^,^29^. However, most of these attempts are time consuming and not cost effective^28^. In this scenario, plant-associated microbes with the capacity to improve drought tolerance in crop plants have received increasing attention^3^,^10^,^12^. Rhizospheric and endophytic bacteria isolated from date palm, grapevine, olive trees and pepper plants cultivated under desert farming conditions have been shown to improve the growth of different host species under water stress conditions^4^,^7^,^9^,^30^. *Pseudomonas putida* MTCC5279, a PGP rhizobacterium isolated from the desert regions of Rajasthan, was able to increase drought resistance in chickpea^11^. The bacterial endophyte *Bacillus subtilis* strain B26, isolated from leaf blades and seeds of switchgrass, was capable of increasing drought resistance in both *Brachypodium distachyon* and Timothy^31^,^32^. Other PGP bacteria with the capacity to improve drought tolerance in crop plants such as wheat, maize, tomato and beans have also been identified^30^,^33^,^34^. In our study, *A. sparsifolia*, the dominant vegetation in the extreme drought region of Taklamakan Desert in north-west China, was selected to discover unique microbial resources with potential to enhance plant drought resistance.

Strain LTYR-11Z^T^ was isolated from surface-sterilized *A. sparsifolia* leaves and the polyphasic approach revealed that this strain merits assignment to a novel species of the genus *Pantoea*. Although originally known as a plant pathogen, *Pantoea* is also recognized for its use for plant growth promotion and phytopathogen control^22^,^25^,^35^. Strain LTYR-11Z^T^, a new member of the genus *Pantoea*, was found to exhibit multiple PGP properties, including mineral phosphate solubilization, production of IAA, siderophores, EPS, protease and ammonia, which may facilitate plant growth directly, indirectly or synergistically^4^,^9^,^10^. Moreover, strain LTYR-11Z^T^ was able to grow in the presence of abiotic stresses typically associated with drought, such as salinization (9% NaCl), osmotic stress (20% PEG) and high temperature (48 °C), suggesting that this bacterium can be active and hence express its PGP features *in vivo* under drought conditions. Although most of PGP bacteria capable of increasing plant drought resistence has been reported to produce ACC deaminase^7^,^9^,^11^, strain LTYR-11Z^T^ did not show ACC deaminase activity, suggesting that ACC deaminase is not a prerequisite for bacteria-mediated drought tolerance in plants.

Efficient root colonization by inoculated bacteria is a critical step in the interaction between beneficial bacteria and the host plants^1^,^36^. Some PGP bacteria were already reported to colonize the roots of a variety of plant species, including both monocots and dicot crop species^3^,^30^. For instance, the bacterial endophyte *Burkholderia phytofirmans* PsJN^T^, isolated from the onion roots, was capable of colonize the roots of a wide range of genetically unrelated plant species, such as *Arabidopsis,* tomato, grapevines, maize, potatoes and switchgrass^1^,^6^,^34^,^37^. Although strain LTYR-11Z^T^ was originally identified as an endophyte from surface-sterilized *A. sparsifolia* leaves, confocal microscopy analysis of GFP-tagged LTYR-11Z^T^ showed that this bacterium is a versatile root colonizer, which is capable of efficiently colonizing the rhizoplane of both *Arabidopsis* and wheat. Furthermore, our re-isolation experiments from surface-sterilized roots of the LTYR-11Z^T^ inoculated wheat plants cultivated in soil revealed this bacterium to be a root endophyte. It is possible that strain LTYR-11Z^T^ originally colonizes the roots of *A. sparsifolia* and then translocates to other internal plant tissues such as stems and leaves in the natural habitat.

Some PGP bacteria were reported to promote plant growth under both non-stressed and drought conditions^32^, while others were specifically able to enhance plant growth under drought conditions, but ineffective in plant growth promotion under optimal irrigation conditions^9^. These findings suggest that the PGP activity of bacteria may be either stress-dependent or stress-independent. Our results demonstrate the effectiveness of strain LTYR-11Z^T^ in promoting the growth of wheat plants under both normal and water stress conditions. However, the plant growth parameters between LTYR-11Z^T^-treated plants and the non-inoculated control plants varied more significantly under water stress than under non-stressed conditions. For instance, there were no observable differences in the shoot length of inoculated plants and the non-inoculated control plants under non-stressed conditions, whereas the shoot length of water-stressed plants inoculated with LTYR-11Z^T^ was 17.1% higher than in stressed but non-inoculated plants. Moreover, after 7 days without watering, the control plants showed more severe symptoms of wilting leaves than the inoculated plants. Our findings potentially indicate that strain LTYR-11Z^T^ plays an more important role in the development and health of wheat plants when they are exposed to drought stress.

In agreement with previous studies^11^,^38^, we observed a dramatic increase in proline concentration in leaves of non-inoculated plants in response to drought stress. However, inoculation with strain LTYR-11Z^T^ had the reverse effect to decrease proline concentration under both water stress and non-stressed conditions. Similar result has been reported by Gagné-Bourque *et al*.^32^, who have shown that drought-stressed timothy colonized with the PGP bacterium *Bacillus subtilis* B26 accumulated a smaller amount of proline than did the stressed but non-inoculated plants. While accumulation of proline is a widespread plant response to environmental stresses, it is still controversial if its accumulation is a symptom of stress damages or an indication of stress tolerance^39^. A decrease in proline concentration in the LTYR-11Z^T^-treated plants could thus be indicative that there is less damage in wheat plants in the presence of this bacterium. MDA is the final product of lipid peroxidation and its level can reflect the degree of cell membrane damage^38^. Similarly, wheat plants colonized with strain LTYR-11Z^T^ had significantly lower MDA contents compared with the non-inoculated control in both non-stressed and drought-stressed groups, revealing that inoculation with LTYR-11Z^T^ helps in overcoming membrane damage. Soluble sugars are thought to play a role in drought tolerance by maintaining osmotic turgor^32^. Soluble sugar concentrations have been reported to drastically increase in plant leaves under drought stress^31^,^32^. As expected, level of soluble sugars was significantly elevated in drought-stressed plants, while the presence of strain LTYR-11Z^T^ further increased the concentration, suggesting that strain LTYR-11Z^T^ contributes to increase the accumulation of sugars to allow for better osmotic adjustment and thus reduces drought-induced damage in host plant. Chlorophyll loss has been reported to be associated with drought stress and chlorophyll concentration is accepted as an important indicator of drought tolerance in plants^11^,^40^. Our study revealed that drought reduced chlorophyll content in the leaves, while significantly increase in chlorophyll concentration was observed in LTYR-11Z^T^-treated plants compared to non-inoculated plants under both non-stressed and water stress conditions, suggesting that strain LTYR-11Z^T^ leads to increased biosynthesis of chlorophyll and therefore helps wheat adapt to drought stress. Taken together, our data provide physiological evidence for the positive effects of strain LTYR-11Z^T^ on wheat seedlings under drought stress.

In summary, the present study reports on the isolation, identification and utilization of a novel endophytic bacterium from the leaves of *A. sparsifolia* for plant growth promotion and alleviation of drought stress. On the basis of data from phenotypic, phylogenetic and DNA–DNA relatedness studies, the isoalte LTYR-11Z^T^ is considered to represent a novel species of the genus *Pantoea*, for which the name *Pantoea alhagi* sp. nov. is proposed. This bacterium exhibits multiple PGP traits and tolerates high temperature, salt and osmotic stress. Strain LTYR-11Z^T^ was found to efficiently colonize the wheat root system, promote plant growth and enhance plant tolerance against water stress. The versatility of strain LTYR-11Z^T^ in root colonization, PGP activity and promotion of drought resistance suggests that the drought-protective behavior of this bacterium likely occurs naturally in the desert ecosystem and it is conceivable that such protecting activity can be performed in field conditions. Future studies will need to focus on the molecular mechanisms involved in LTYR-11Z^T^-induced drought tolerance in wheat.

### Description of *Pantoea alhagi* sp. nov

#### *Pantoea alhagi* (al.ha′gi. N.L. gen. n. *alhagi* of the plant genus *Alhagi*)

Cells are Gram-negative, facultatively anaerobic, rod-shaped and motile via peritrichous flagella. Colonies grown on TSA at 30 °C for 24 h are yellow-pigmented, circular and convex with a diameter of approximately 1–2 mm. Growth occurs at 7–48 °C (optimum, 37 °C), at pH 5.0–9.0 (optimum, pH 7.0) and in the presence of 0–9% (w/v) NaCl (optimum, 0–2%). Oxidase-negative and catalase-positive. Hydrolyses aesculin, gelatin, casein and carboxymethyl cellulose, but not starch, tyrosine or chitin. Positive for nitrate reduction, indole production and Voges–Proskauer reaction (acetoin production), and arginine dihydrolase, urease, lysine decarboxylase and ornithine decarboxylase activities. Negative for H_2_S production and tryptophan deaminase activity. In the API 20NE system, positive for assimilation of D-glucose, L-arabinose, D-mannose, D-mannitol, *N*-acetylglucosamine, maltose, potassium gluconate and malic acid, but negative for assimilation of capric acid, adipic acid, trisodium citrate and phenylacetic acid. In the API 50CHE system, acid is produced from L-arabinose, D-ribose, D-xylose, D-galactose, D-glucose, D-fructose, D-mannose, L-rhamnose, inositol, D-mannitol, D-sorbitol, *N*-acetylglucosamine, amygdalin, aesculin, D-cellobiose, D-maltose, D-melibiose, sucrose, D-trehalose, gentiobiose, and produced weakly from glycerol, arbutin and D-lactose, but not produced from erythritol, D-arabinose, L-xylose, D-adonitol, methyl *β*-D-xylopyranoside, L-sorbose, dulcitol, methyl *α*-D-mannopyranoside, methyl *α*-D-glucopyranoside, salicin, inulin, D-melezitose, D-raffinose, starch, glycogen, xylitol, D-turanose, D-lyxose, D-tagatose, D-fucose, L-fucose, D-arabitol, L-arabitol, potassium gluconate, potassium 2-ketogluconate or potassium 5-ketogluconate. In the API ZYM system, positive for alkaline phosphatase, leucine arylamidase, acid phosphatase, naphthol-AS-BI-phosphohydrolase and *β*-galactosidase, weakly positive for valine arylamidase, trypsin and *β*-glucosidase and negative for esterase (C4), esterase lipase (C8), lipase (C14), cystine arylamidase, *α*-chymotrypsin, *α*-galactosidase, *β*-glucuronidase, *α*-glucosidase, *N*-acetyl-*β*-glucosaminidase, *α*-mannosidase and *α*-fucosidase. The predominant cellular fatty acids are C_16:0_, summed feature 3 (comprising C_16:1_*ω*7*c* and/or C_16:1_*ω*6*c*), C_17:0_ cyclo and summed feature 2 (comprising any combination of C_12:0_ aldehyde, an unknown fatty acid of equivalent chain length 10.928, iso-C_16:1_ I and C_14:0_ 3-OH). The DNA G+C content of the type strain is 53.4 mol%.

The type strain, LTYR-11Z^T^ (=CCTCC M 2016052^T^=KCTC 52262^T^), was isolated from surface-sterilized leaves of *Alhagi sparsifolia* collected from Taklamakan Desert in Xinjiang Uyghur Autonomous Region, north-west China. The 16S rRNA, ATP synthase beta subunit (*atpD*), DNA gyrase B subunit (*gyrB*), initiation translation factor 2 (*infB*) and RNA polymerase beta subunit (*rpoB*) gene sequences of strain LTYR-11Z^T^ have been deposited in GenBank/EMBL/DDBJ under the accession numbers KX494924-KX494928, respectively.

## Methods

### Isolation and maintenance of the isolate

Apparently healthy plant samples of *Alhagi sparsifolia* were collected in August 2014 from Taklamakan Desert, Xinjiang Uyghur Autonomous Region, north-west China. The plant samples were carefully washed under tap water to remove surface soil and the leaves, stems and roots were separated. After drying at room temperature, the tissue segments were subjected to a surface sterilization procedure as described by Zhang *et al*.^41^. The efficacy of the sterilization method was verified by plating the last wash water and by placing pieces of the sterilized tissues on TSA. The surface-sterilized samples were cut into small fragments, macerated using a sterile pestle and mortar, serially diluted in sterile distilled water and spread-plated onto R2A agar supplemented with 50 μg cycloheximide ml^−1^. After incubation at 28 °C for 1 week, bacterial colonies were picked up and further purified by repeated streaking on the same medium. LTYR-11Z^T^ was routinely cultivated on TSA at 30 °C and long term storage was performed via cryopreservation with 20% (v/v) glycerol at −80 °C.

### 16S rRNA gene sequencing and phylogenetic analysis

The universal primers 27f and 1492r^42^ were used for PCR amplification of the 16S rRNA gene of LTYR-11Z^T^. The resulting 16S rRNA gene sequence was compared against 16S rRNA gene sequences available from the EzTaxon database (http://eztaxon-e.ezbiocloud.net/)^43^ and the GenBank database (http://blast.ncbi.nlm.nih.gov/Blast/). Multiple sequence alignments were performed with CLUSTAL X^44^. Phylogenetic trees were reconstructed with the NJ and ML methods in the software package MEGA version 5.1^45^. The distance matrix was constructed according to the Kimura two-parameter model^46^. The resultant tree topologies were evaluated by bootstrap analyses based on 1000 resamplings.

### MLSA analysis

Partial *atpD, gyrB, infB* and *rpoB* genes of LTYR-11Z^T^ were amplified and sequenced following the protocol of Brady *et al*.^19^. The resulting gene sequences were aligned with those from the closely related species of genera *Erwinia, Pantoea* and *Tatumella* by using the CLUSTAL X software^44^. NJ and ML phylogenetic trees based on the concatenated partial *atpD, gyrB, infB* and *rpoB* gene sequences were reconstructed using the same methods as described above.

### Determination of DNA G+C content of LTYR-11Z^T^ and DNA–DNA hybridization

The genomic DNA of LTYR-11Z^T^ and the two type strains *P. theicola* NBRC 110557^T^ and *P. intestinalis* DSM 28113^T^ was prepared as described previously^41^. The genomic DNA of LTYR-11Z^T^ was treated with nuclease P1 and alkaline phosphatase and its DNA base composition was determined by reverse-phase HPLC as described by Mesbah *et al*.^47^. DNA–DNA hybridization was carried out by using photobiotin-labelled probes in microplate wells as described by Ezaki *et al*.^48^. Hybridization was performed at 43 °C in the presence of 50% formamide, with five replications for each sample. DNA–DNA hybridization values are presented as means ± standard deviations.

### Phenotypic Characteristics

Cell morphology was examined by transmission electron microscopy (Hitachi HT7700) and phase contrast microscopy (BX51 microscope; Olympus) with cells grown on TSA at 30 °C for 24 h. Gram staining was performed as described previously^49^. Motility was determined using semi-solid agar as described by Lin *et al*.^50^. The growth temperature range (4, 7, 10, 15, 25, 28, 30, 37, 42, 48 and 52 °C) and NaCl tolerance [0–10% (w/v) NaCl at 1% intervals] were tested on NA for 7 days. The pH range and optimum for growth was tested in TSB adjusted to pH 4.0–11.0 at intervals of 0.5 pH units using the buffer system as described previously^42^. Anaerobic growth was tested on TSA at 30 °C for 7 days in an anaerobic jar by using AnaeroGen anaerobic system envelopes (Oxoid). Oxidase and catalase activities and hydrolysis of carboxymethyl cellulose, casein, chitin and starch were determined on TSA plates following protocols described by Zhang *et al*.^41^. Antibiotic resistance was tested with ampicillin, chloramphenicol, kanamycin, neomycin sulfate, streptomycin, tetracycline and vancomycin at concentrations of 5, 50, 100 and 300 μg ml^−1^ using TSB medium. Carbon-source utilization, activities of constitutive enzymes and other physiological properties were determined by using the API 20E, API 20NE, API 50CHE and API ZYM systems (bioMérieux) according to the manufacturer’s instructions. The two closely related type strains, *P. theicola* NBRC 110557^T^ and *P. intestinalis* DSM 28113^T^, were grown under the same conditions and used as reference strains for phenotypic characterization.

### Fatty acid analysis

For whole-cell fatty acid analysis, LTYR-11Z^T^ and the two reference strains *P. theicola* NBRC 110557^T^ and *P. intestinalis* DSM 28113^T^ were grown on TSA at 30 °C for 24 h and fresh cells of comparable physiological age were harvested from the third streak quadrant of the agar plates. Cellular fatty acids were saponified, methylated and extracted according to the standard protocol of the MIDI Sherlock Microbial Identification System (version 6.0). Fatty acid methyl esters were analysed by gas chromatography (6890N; Hewlett Packard) and identified using the TSBA6 database of the Microbial Identification System^51^.

### *In vitro* characterization of the PGP potential and abiotic stress tolerance of LTYR-11Z^T^

IAA production was assessed by the Salkowski assay as described by Gutierrez *et al*.^52^. The mineral P-solubilizing ability of LTYR-11Z^T^ was first assessed on NBRIP agar plates containing 0.5% Ca_3_(PO_4_)_2_^53^. Quantitative estimation of phosphate solubilization was performed in NBRIP liquid medium using the Molybdenum-blue method^54^. Siderophore production was determined by using chrome azurol S agar plates^55^. EPS production was estimated on RCV mineral medium supplemented with 0.01% yeast extract and 4% (w/v) sucrose^56^. Ammonia production was tested in peptone water as described previously^7^. ACC deaminase activity was determined according to the protocol described by Penrose and Glick^57^. Tolerance to osmotic stress was evaluated by adding 10–20% PEG to the liquid media and incubating in 30 °C for 3 days.

### Construction of GFP-labeled LTYR-11Z^T^

The *gfp* reporter gene was amplified from plasmid pKEN-GFPmut3*^58^ using the primer pair gfpmut3-F (5′-ACATGCATGCATGAGTAAAGGAGAAGAACTTTTCACT-3′) and gfpmut3-F (5′-CTAGTCTAGATTATTTGTATAGTTCATCCATGCCA-3′). The PCR product was digested with *Sph*I/*Xba*I and inserted into the *Sph*I/*Xba*I sites of pKT100 to generate pKT100-GFPmut3*. The pKT100-GFPmut3* plasmid was electroporated into LTYR-11Z^T^ as described by Marasco *et al*.^7^. The GFP-labelled LTYR-11Z^T^ was selected against kanamycin (50 μg ml^−1^) and checked using a fluorescence microplate reader (Gemini XPS; Molecular Device, USA).

### Root colonization assay

Seeds of *Arabidopsis thaliana* and winter wheat (cultivar Yumai 49–198) were surface sterilized with 75% ethanol for 30 s and then 0.1% HgCl_2_ for 7 min followed by five rinses in sterile distilled water. Surface-sterilized *A. thaliana* seeds were kept at 4 °C for 3 days to synchronize germination and then sown on half-strength Murashige and Skoog (MS) medium^59^ 0.8% agar plates supplemented with 1% sucrose. Plates were placed in a growth chamber at 22 °C with a photoperiod of 16 h of light and 8 h of dark. Surface-sterilized wheat seeds were transferred to damp filter paper in Petri dishes and germinated at 25 °C in the dark. Three-day-old *A. thaliana* seedlings and two-days-old wheat seedlings were separately dipped in sterile saline solution (8.5 g NaCl l^−1^) containing kanamycin (50 μg ml^−1^) and 10^8^ cells ml^−1^ bacterial suspension of the GFP-labelled LTYR-11Z^T^. Seedlings dipped in saline solution without bacterial inoculation were used as negative control. After 2, 5 and 15 h of incubation, plant roots were gently washed to remove weakly bound bacteria and observed using a laser scanning confocal microscope (Leica, Germany) with excitation at 488 nm and emission at 520 nm.

### Plant growth promotion in soil under non-stressed and water stress conditions

The surface-sterilized wheat seeds were germinated for 2 days at 25 °C in the dark. The uniform-sized seedlings were selected and planted in sterilized field soil, three plants per 14-cm plastic pot. The pots were transferred to a growth chamber at 25 °C with a photoperiod of 16/8 h light/dark. After one week, the seedlings were inoculated with the bacterial suspension of LTYR-11Z^T^ in sterilized tap water at the concentration of 10^8^ cells g^−1^ of soil, while non-inoculated seedlings were watered with sterilized tap water. One week after bacteria inoculation, watering was terminated for 7 days. A positive control was properly irrigated throughout the experiment. After drought treatment, water irrigation was resumed for 1 day, and plant health was assessed and photographed. Then plants of different treatments were harvested for biomass and length measurements. Leaves from each treatment were respectively collected and used for measurements of free proline, soluble sugars, MDA and total chloropyhll as described previously^11^,^38^. For the analysis of endophytic colonization, the roots of three plants per treatment were surface sterilized, cut into small pieces and macerated in sterile mortars. The homogenized tissue was serially diluted in sterile saline solution and plated onto TSA containing 100 μg ampicillin ml^−1^. The number of CFU per gram of root were determined after 48 h of incubation at 30 °C. Three independent experiments were performed with 9 individual plants per treatment in each experiment. Statistical analysis was performed by one-way ANOVA with Brown-Forsythe test and a difference was considered statistically significant when P < 0.05.

## Additional Information

**How to cite this article:** Chen, C. *et al*. *Pantoea alhagi*, a novel endophytic bacterium with ability to improve growth and drought tolerance in wheat. *Sci. Rep.* **7**, 41564; doi: 10.1038/srep41564 (2017).

**Publisher's note:** Springer Nature remains neutral with regard to jurisdictional claims in published maps and institutional affiliations.

## Supplementary Material

Supplementary Information

## Figures and Tables

**Table 1:** Differential characteristics of strain LTYR-11Z^T^ and type strains of phylogenetically related species of the genus *Pantoea.*

Characteristic	1	2	3
Colony colour on TSA	Yellow	White	Cream
Indole production	+	−	−
Acetoin production	+	−	+
Urease	+	+	−
Growth at/in:			
pH 4.5	−	−	+
45 °C	+	−	+
9% NaCl	+	−	+
Hydrolysis of:			
Casein (protease)	+	−	−
Gelatin (gelatinase)	+	−	−
Resistant to Ampicillin (50 and 100 μg ml^−l^)	+	−	+
Assimilation of malic acid (API 20NE)	+	−	+
Enzyme activities (API ZYM)			
Alkaline phosphatase	+	+	−
*α*-Glucosidase	−	+	−
*β*-Glucosidase	+	+	−
*N*-Acetyl-*β*-glucosaminidase	−	(+)	−
Acid production from (API 50CHE):			
Amygdalin	+	(+)	−
D-Sorbitol	+	−	−
Sucrose	+	−	−
D-Lactose	(+)	−	+
Salicin	−	+	−
D-Fucose	−	+	+

Strains: 1, LTYR-11Z^T^; 2, *Pantoea theicola* NBRC 110557^T^; 3, *Pantoea intestinalis* DSM 28113^T^. All data were obtained from this study. +, Positive; -, negative; (+), weak. All strains are positive for growth on LB agar, R2A agar, NA and TSA and MacConkey agar, nitrate reduction, hydrolysis of aesculin, carboxymethyl cellulose and 4-nitrophenyl-*β*-D*-*galactopyranoside (PNPG), catalase, arginine dihydrolase, leucine arylamidase, valine arylamidase, trypsin, acid phosphatase, naphthol-AS-BI-phosphohydrolase and *β*-galactosida se, and assimilation of D-glucose, L-arabinose, D-mannose, D-mannitol, *N*-acetylglucosamine, maltose and potassium gluconate. All strains are negative for hydrolysis of L-tyrosine, starch and chitin, H_2_S production, oxidase, tryptophan deaminase, esterase (C4), esterase lipase (C8), lipase (C14), cystine arylamidase, *α*-chymotrypsin, *α*-galactosidase, *β*-glucuronidase, *α*-mannosidase and *α*-fucosidase and assimilation of capric acid, adipic acid, trisodium citrate and phenylacetic acid.

**Table 2:** Cellular fatty acid profiles of strain LTYR-11Z^T^ and type strains of closely related species of the genus *Pantoea.*

Fatty acid	1	2	3
C_12:0_	8.8	5.9	4.2
C_13:0_	0.1	—	0.3
C_14:0_	6.3	8.4	6.5
C_16:0_	25.3	32.5	34.9
C_17:0_ cyclo	11.9	10.8	15.2
C_17:0_	—	—	0.4
C_16:0_ 3-OH	0.5	—	—
C_18:1_*ω*9*c*	0.6	—	—
C_18:0_	0.8	1.2	1.0
C_18:1_*ω*7c 11-methyl	—	—	0.3
iso-C_17:0_ 3-OH	3.4	—	—
iso-C_19:0_	—	2.5	—
C_19:0_ cyclo *ω*8c	—	—	3.3
Summed features			
2	11.0	9.0	12.2
3	21.8	15.4	8.6
8	9.5	14.3	13.1

Strains: 1, LTYR-11Z^T^; 2, *Pantoea theicola* NBRC 110557^T^; 3, *Pantoea intestinalis* DSM 28113^T^. All data were obtained from this study. Values are percentages of the total fatty acids; −, not detected. Summed features are combinations of fatty acids that cannot be separated by the MIDI system. Summed feature 2 comprises any combination of C_12:0_ aldehyde, an unknown fatty acid of equivalent chain length 10.928, iso-C_16:1_ I and C_14:0_ 3-OH; summed feature 3 comprises C_16:1_*ω*7*c* and/or C_16:1_*ω*6*c*; summed feature 8 comprises C_18:1_ω7c and/or C_18:1_ω6c.
