# Author Correction: *Pantoea alhagi*, a novel endophytic bacterium with ability to improve growth and drought tolerance in wheat

**DOI:** 10.1038/s41598-021-86899-4

**Published:** 2021-04-09

**Authors:** Chaoqiong Chen, Kaiyun Xin, Hao Liu, Juanli Cheng, Xihui Shen, Yao Wang, Lei Zhang

**Affiliations:** 1 State Key Laboratory of Crop Stress Biology for Arid Areas and College of Life Sciences, Northwest A&F University, Yangling, Shaanxi 712100 PR China; 2 Life Sciences Department, Yuncheng University, Yuncheng, 044000 PR China

Correction to: *Scientific Reports* 10.1038/srep41564, published online 27 January 2017

The Article contains an error in the Discussion section under subheading ‘Description of *Pantoea alhagi*sp. nov’

‘The type strain, LTYR-11Z^T^ (=CCTCC M 2016052^T^=KCTC 52262^T^), was isolated from surface-sterilized leaves of *Alhagi sparsifolia* collected from Taklamakan Desert in Xinjiang Uyghur Autonomous Region, north-west China. The 16S rRNA, ATP synthase beta subunit (*atpD*), DNA gyrase B subunit (*gyrB*), initiation translation factor 2 (*infB*) and RNA polymerase beta subunit (*rpoB*) gene sequences of strain LTYR-11Z^T^ have been deposited in GenBank/EMBL/DDBJ under the accession numbers KX494924-KX494928, respectively.’

should read:

‘The type strain, LTYR-11Z^T^ (=CCTCC AB 2019082^T^=KCTC 52262^T^), was isolated from surface-sterilized leaves of *Alhagi sparsifolia* collected from Taklamakan Desert in Xinjiang Uyghur Autonomous Region, north-west China. The 16S rRNA, ATP synthase beta subunit (*atpD*), DNA gyrase B subunit (*gyrB*), initiation translation factor 2 (*infB*) and RNA polymerase beta subunit (*rpoB*) gene sequences of strain LTYR-11Z^T^ have been deposited in GenBank/EMBL/DDBJ under the accession numbers KX494924-KX494928, respectively.’

