# Supplementary material for: Pantoea alhagi, a novel endophytic bacterium with ability to improve growth and drought tolerance in wheat
Source: Sci Rep. 2017 Jan 27;7:41564. doi: 10.1038/srep41564 (PMC5269684; doi:10.1038/srep41564)

Pantoea alhagi, a novel endophytic bacterium with ability to improve growth and drought tolerance in wheat

Chaoqiong Chen[1]†, Kaiyun Xin[1]†, Hao Liu[1], Juanli Cheng[1, 2], Xihui Shen[1], Yao Wang[1] & Lei Zhang[1]*

[1] State Key Laboratory of Crop Stress Biology for Arid Areas and College of Life Sciences, Northwest A&F University, Yangling, Shaanxi 712100, PR China

[2] Life Sciences Department, Yuncheng University, Yuncheng 044000, PR China

† These authors contributed equally to this work.

*Corresponding Author: Lei Zhang, E-mail: zhanglei0075@nwsuaf.edu.cn

Tel: +86-29-87092087; Fax: +86-29-87092087.

Fig. S1. Maximum-likelihood phylogenetic tree based on 16S rRNA gene sequences showing the relationships between strain LTYR-11Z^{T} and related type strains of the genus *Pantoea*, *Erwinia* and *Tatumella*. Numbers at nodes indicate bootstrap percentages (based on 1000 resampled datasets), and only values above 50% are shown. *Brenneria alni* ICMP 12481^{T} was used as an outgroup. Bar, 0.02 substitutions per nucleotide position.

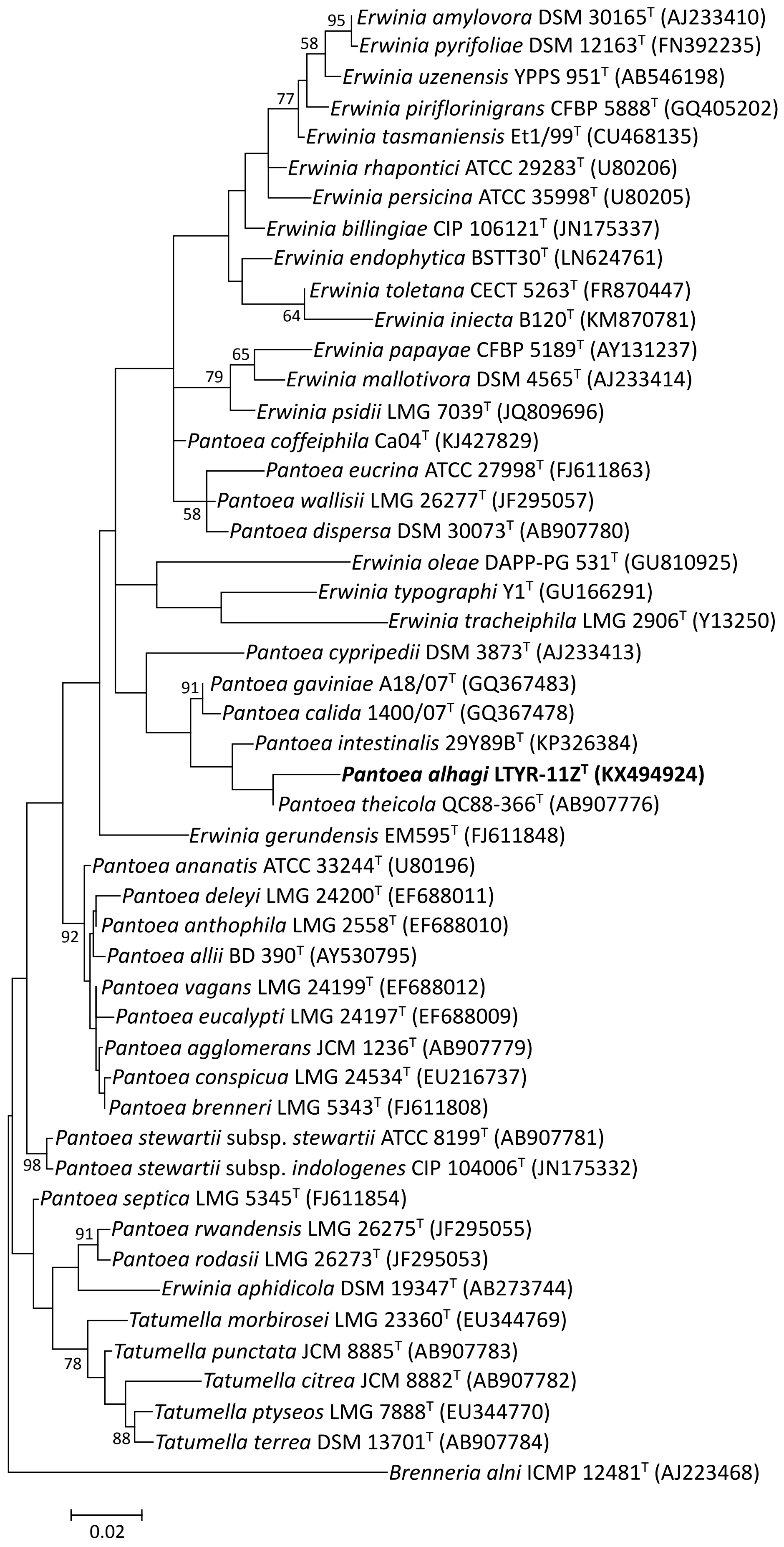
95
Erwinia amylovora DSM 30165^{T} (AJ233410)
58
Erwinia pyrifoliae DSM 12163^{T} (FN392235)
Erwinia uzenensis YPPS 951^{T} (AB546198)
77
Erwinia piriflorinigrans CFBP 5888^{T} (GQ405202)
Erwinia tasmaniensis Et1/99^{T} (CU468135)
Erwinia rhapontici ATCC 29283^{T} (U80206)
Erwinia persicina ATCC 35998^{T} (U80205)
Erwinia billingiae CIP 106121^{T} (JN175337)
Erwinia endophytica BSTT30^{T} (LN624761)
Erwinia toletana CECT 5263^{T} (FR870447)
64
Erwinia iniecta B120^{T} (KM870781)
65
Erwinia papayae CFBP 5189^{T} (AY131237)
79
Erwinia mallotivora DSM 4565^{T} (AJ233414)
Erwinia psidii LMG 7039^{T} (JQ809696)
Pantoea coffeiphila Ca04^{T} (KJ427829)
Pantoea eucrina ATCC 27998^{T} (FJ611863)
Pantoea wallisii LMG 26277^{T} (JF295057)
58
Pantoea dispersa DSM 30073^{T} (AB907780)
Erwinia oleae DAPP-PG 531^{T} (GU810925)
Erwinia typographi Y1^{T} (GU166291)
Erwinia tracheiphila LMG 2906^{T} (Y13250)
Pantoea cypripedii DSM 3873^{T} (AJ233413)
91
Pantoea gaviniae A18/07^{T} (GQ367483)
Pantoea calida 1400/07^{T} (GQ367478)
Pantoea intestinalis 29Y89B^{T} (KP326384)
***Pantoea alhagi* LTYR-11Z^{T} (KX494924)**
Pantoea theicola QC88-366^{T} (AB907776)
Erwinia gerundensis EM595^{T} (FJ611848)
Pantoea ananatis ATCC 33244^{T} (U80196)
Pantoea deleyi LMG 24200^{T} (EF688011)
Pantoea anthophila LMG 2558^{T} (EF688010)
92
Pantoea allii BD 390^{T} (AY530795)
Pantoea vagans LMG 24199^{T} (EF688012)
Pantoea eucalypti LMG 24197^{T} (EF688009)
Pantoea agglomerans JCM 1236^{T} (AB907779)
Pantoea conspicua LMG 24534^{T} (EU216737)
Pantoea brenneri LMG 5343^{T} (FJ611808)
Pantoea stewartii subsp. *stewartii* ATCC 8199^{T} (AB907781)
98
Pantoea stewartii subsp. *indologenes* CIP 104006^{T} (JN175332)
Pantoea septica LMG 5345^{T} (FJ611854)
91
Pantoea rwandensis LMG 26275^{T} (JF295055)
Pantoea rodasii LMG 26273^{T} (JF295053)
Erwinia aphidicola DSM 19347^{T} (AB273744)
Tatumella morbirosei LMG 23360^{T} (EU344769)
78
Tatumella punctata JCM 8885^{T} (AB907783)
Tatumella citrea JCM 8882^{T} (AB907782)
Tatumella ptyseos LMG 7888^{T} (EU344770)
88
Tatumella terrea DSM 13701^{T} (AB907784)
Brenneria alni ICMP 12481^{T} (AJ223468)
0.02

Fig. S2. Maximum-likelihood phylogenetic tree based on concatenated partial sequences of *atpD*, *gyrB*, *infB* and *rpoB* gene sequences showing the relationships between strain LTYR-11Z^T and related species of the genus *Pantoea*, *Erwinia* and *Tatumella*. Numbers at nodes indicate bootstrap percentages (based on 1000 resampled datasets), and only values above 50% are shown. *Brenneria alni* NCPPB 3934^T was used as an outgroup. Bar, 0.05 substitutions per nucleotide position.

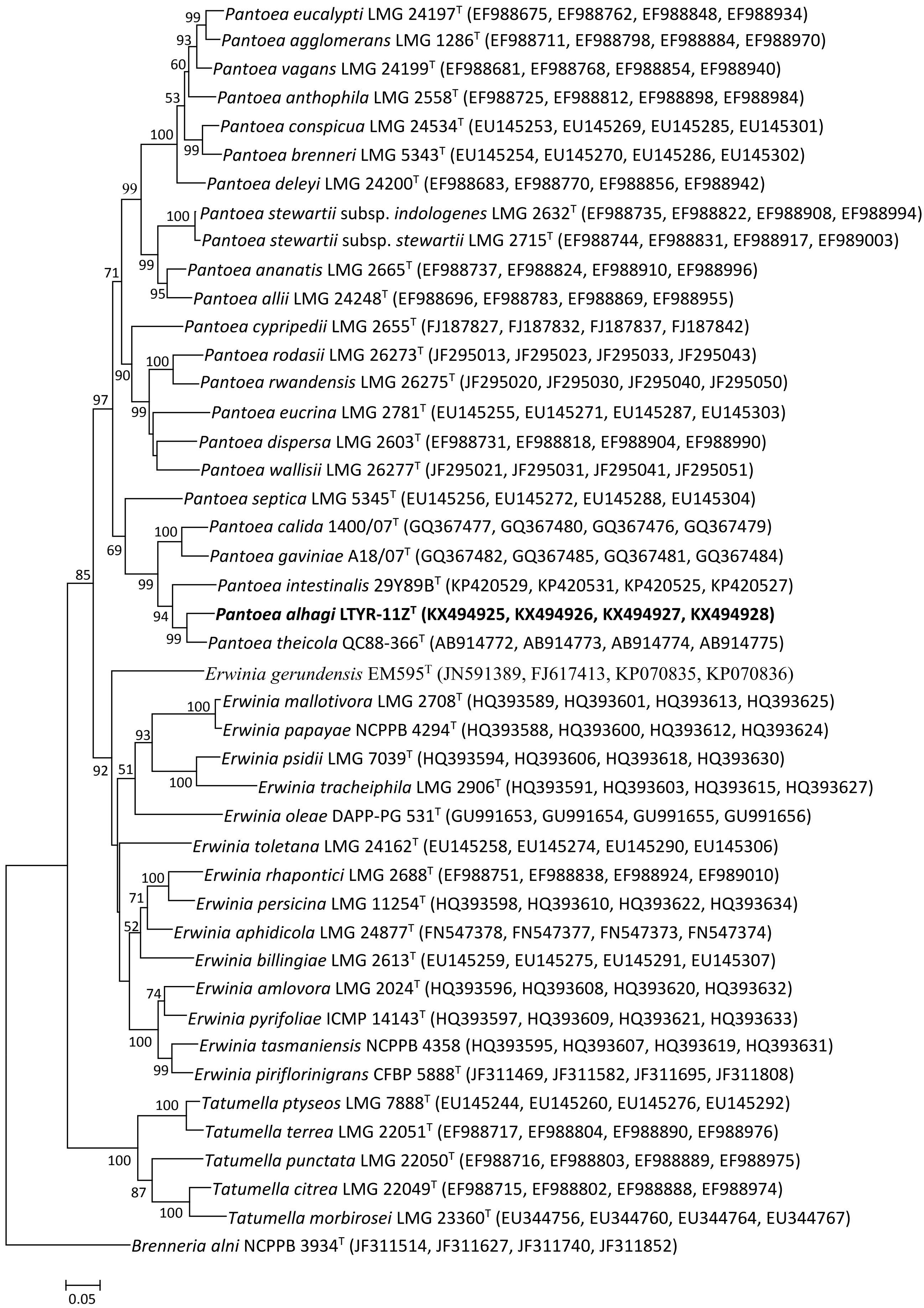
Pantoea eucalypti LMG 24197^{T} (EF988675, EF988762, EF988848, EF988934)
Pantoea agglomerans LMG 1286^{T} (EF988711, EF988798, EF988884, EF988970)
Pantoea vagans LMG 24199^{T} (EF988681, EF988768, EF988854, EF988940)
Pantoea anthophila LMG 2558^{T} (EF988725, EF988812, EF988898, EF988984)
Pantoea conspicua LMG 24534^{T} (EU145253, EU145269, EU145285, EU145301)
Pantoea brenneri LMG 5343^{T} (EU145254, EU145270, EU145286, EU145302)
Pantoea deleyi LMG 24200^{T} (EF988683, EF988770, EF988856, EF988942)
Pantoea stewartii subsp. indologenes LMG 2632^{T} (EF988735, EF988822, EF988908, EF988994)
Pantoea stewartii subsp. stewartii LMG 2715^{T} (EF988744, EF988831, EF988917, EF989003)
Pantoea ananatis LMG 2665^{T} (EF988737, EF988824, EF988910, EF988996)
Pantoea allii LMG 24248^{T} (EF988696, EF988783, EF988869, EF988955)
Pantoea cypripedii LMG 2655^{T} (FJ187827, FJ187832, FJ187837, FJ187842)
Pantoea rodasii LMG 26273^{T} (JF295013, JF295023, JF295033, JF295043)
Pantoea rwandensis LMG 26275^{T} (JF295020, JF295030, JF295040, JF295050)
Pantoea eucrina LMG 2781^{T} (EU145255, EU145271, EU145287, EU145303)
Pantoea dispersa LMG 2603^{T} (EF988731, EF988818, EF988904, EF988990)
Pantoea wallisii LMG 26277^{T} (JF295021, JF295031, JF295041, JF295051)
Pantoea septica LMG 5345^{T} (EU145256, EU145272, EU145288, EU145304)
Pantoea calida 1400/07^{T} (GQ367477, GQ367480, GQ367476, GQ367479)
Pantoea gaviniae A18/07^{T} (GQ367482, GQ367485, GQ367481, GQ367484)
Pantoea intestinalis 29Y89B^{T} (KP420529, KP420531, KP420525, KP420527)
Pantoea alhagi LTYR-11Z^{T} (KX494925, KX494926, KX494927, KX494928)
Pantoea theicola QC88-366^{T} (AB914772, AB914773, AB914774, AB914775)
Erwinia gerundensis EM595^{T} (JN591389, FJ617413, KP070835, KP070836)
Erwinia mallotivora LMG 2708^{T} (HQ393589, HQ393601, HQ393613, HQ393625)
Erwinia papayae NCPPB 4294^{T} (HQ393588, HQ393600, HQ393612, HQ393624)
Erwinia psidii LMG 7039^{T} (HQ393594, HQ393606, HQ393618, HQ393630)
Erwinia tracheiphila LMG 2906^{T} (HQ393591, HQ393603, HQ393615, HQ393627)
Erwinia oleae DAPP-PG 531^{T} (GU991653, GU991654, GU991655, GU991656)
Erwinia toletana LMG 24162^{T} (EU145258, EU145274, EU145290, EU145306)
Erwinia rhapontici LMG 2688^{T} (EF988751, EF988838, EF988924, EF989010)
Erwinia persicina LMG 11254^{T} (HQ393598, HQ393610, HQ393622, HQ393634)
Erwinia aphidicola LMG 24877^{T} (FN547378, FN547377, FN547373, FN547374)
Erwinia billingiae LMG 2613^{T} (EU145259, EU145275, EU145291, EU145307)
Erwinia amlovora LMG 2024^{T} (HQ393596, HQ393608, HQ393620, HQ393632)
Erwinia pyrifoliae ICMP 14143^{T} (HQ393597, HQ393609, HQ393621, HQ393633)
Erwinia tasmaniensis NCPPB 4358 (HQ393595, HQ393607, HQ393619, HQ393631)
Erwinia piriflorinigrans CFBP 5888^{T} (JF311469, JF311582, JF311695, JF311808)
Tatumella ptyseos LMG 7888^{T} (EU145244, EU145260, EU145276, EU145292)
Tatumella terrea LMG 22051^{T} (EF988717, EF988804, EF988890, EF988976)
Tatumella punctata LMG 22050^{T} (EF988716, EF988803, EF988889, EF988975)
Tatumella citrea LMG 22049^{T} (EF988715, EF988802, EF988888, EF988974)
Tatumella morbirosei LMG 23360^{T} (EU344756, EU344760, EU344764, EU344767)
Brenneria alni NCPPB 3934^{T} (JF311514, JF311627, JF311740, JF311852)
0.05

Fig. S3. Transmission electron micrograph showing the general morphology of negatively stained cells of strain LTYR-11Z^{T} after growth for 24 h at 30 °C on TSA. Bar, 500 nm.

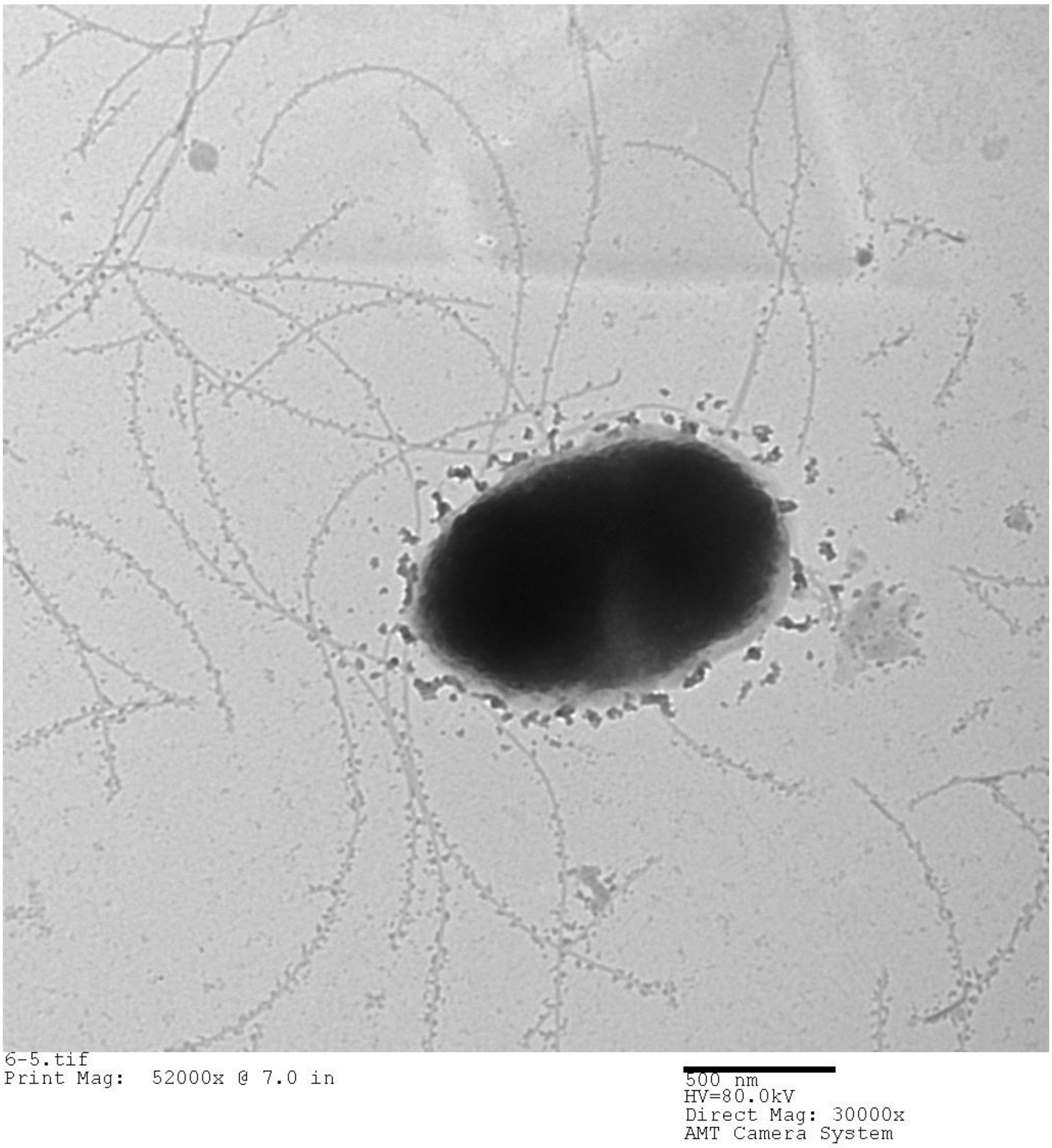

Supplement: Supplementary Information [file srep41564-s1.pdf]
